# On the Use of the Oil of Turpentine in Iritis

**Published:** 1832-01

**Authors:** John Foote


### IRITIS.

### *On the Use of the Oil of Turpentine in Iritis.*

By John Foote, Jun., Esq.

(Read before the Western Medico-Chirurgical Debating Society.)

Inflammation of the iris may exist independent of, and totally unconnected with, inflammation in any other membrane of the eye, such as sclerotitis or corneitis: such is the opinion of Mr. Mackenzie, of Glasgow: yet it is more frequently accompanied with one or more of these inflammations. Many medical men have seen cases of iritis with corneitis, sclerotitis, or inflammation of the conjunctiva, that have never seen a case of simple iritis, or one unattended with any other inflammation. From what I have seen of it, its more frequent accompaniment is the inflammation of the conjunctiva; and, from this disease being occasionally severe, the iritis is sometimes overlooked, more especially if the attack be slight. When the inflammation runs very high, it is liable to extend to the choroid coat and retina: this fact has induced some writers to deny that iritis can occur without extending to the retina; but it is a mistake: *simple iritis* may take place. The disease commences most frequently on the pupillary margin of the iris, and extends thence through the whole of the tunic; and so rapid is its progress in the adhesive process, that we sometimes see effusion taking place on the pupillary margin, and the lymph even becoming organized, ere the rest of the iris is much engaged in the disease. It is from the extreme rapidity with which the adhesive process proceeds that iritis is so dangerous, and requires such energetic measures. The case of Ann Lawrence, hereafter mentioned, exemplifies the rapidity of this process; the pupil being irregular in the first attack, ten days after its commencement, though the disease was apparently proceeding slowly; and in the second, the pupil was irregular within three days after the commencement of the disease.

*Semeiology*. The disease is characterized by certain symptoms in all its forms. We have a beautiful zone of pink hair-like vessels running towards the cornea, forming an appearance like the rays of the sun, as represented radiating from that body. A change of colour of the iris is also remarkably evident, owing to its increased vascularity: if blue, it becomes greenish; if dark-coloured, reddish. Contraction, irregularity, and immobility of the pupil speedily follow, unless prevented by proper treatment. Effusion of coagulable lymph into the pupil and posterior chamber, rarely into the anterior; adhesions of the iris; dimness of sight, and sometimes almost total blindness; pain in the eye, and nocturnal circumorbital pain.

This classification of symptoms is according to Mackenzie, in his splendid work on the Eye: all, or the greater part, exist in every case of iritis. The change of colour of the iris, the zonular appearance, irregularity of the pupil, together with the pain, are the most frequent symptoms, and are those also which best indicate the nature of the complaint.

Iritis is frequently divided into idiopathic, traumatic, sympathetic, syphilitic, rheumatic, and arthritic: besides these, there are various compound cases, as when the rheumatic and arthritic are combined, or the rheumatico-syphilitic, or else the arthritico-syphilitic. These distinctions depend more on the causes than on the semiology or treatment of the disease: thus, the idiopathic arises from cold, or some unknown cause; the traumatic, from wounds; the sympathetic, when it supervenes on some other long-continued or very severe inflammation, as on sclerotitis, &c.: these are evident. The rheumatico- or the arthritico-syphilitic depends on syphilitic iritis appearing in persons with a rheumatic or arthritic taint in their constitution; and these cases will generally require the addition of colchicum to the means we employ to effect a cure.

*General treatment.* In strong and plethoric subjects, and more especially in the traumatic, syphilitic, and occasionally the sympathetic, the patient should be bled freely from the arm, *pro ut ferant vires:* this will remove the pain for a time, or at least diminish it very considerably. If the pain return violently, the bleeding must be repeated: but it will generally be found sufficient in that case to bleed locally by cupping, in preference to leeches. The generality of cuppers open the temporal artery in cupping on the temples, and we thus obtain what we wanted, and which we could not get by leeches, a rapid loss of blood from the part: the cupper should be always desired to open the artery, or a branch of it. Next to bloodletting, or rather equal, and by some considered superior to it, ranks Mercury: this is frequently administered alone, without any other remedial agent, and has certainly, under such circumstances, cured the disease, of which I have seen several instances.

The best form for giving this medicine is a combination of calomel and opium: two grains of calomel and a quarter of a grain of opium, (the dose of the latter increased, if purging come on,) given every second, third, or fourth hour, according to the urgency of the case, so as to salivate rapidly. When the disease continues advancing, and it is not easy to produce that effect of mercury by which it manifests its action, inunction on the temple, either of the Ung. Hydrarg. alone or combined with belladonna, may be had recourse to with benefit. The power of mercury over this disease is so very great, that it has been considered a specific; and yet, such is the strange anomaly of medical facts, it is supposed that mercury can produce the disease. I have never seen an instance which I could fairly attribute to mercury; and numerous instances are recorded in which iritis appeared, although no mercury had been given.

In the rheumatic and arthritic, and also in the syphilitic, when combined with these diatheses, it will be necessary to adjoin colchicum, or some of its preparations, with the mercury: in such a case, if calomel be given, it should be administered in conjunction with the extract of colchicum and opium; but the better way of giving mercury is by inunction, and the internal use of the Vinum Colchici. Such subjects do not bear bleeding well, and it is apt sometimes, more especially in bad constitutions, to prove injurious, by inducing a chronic form, which is difficult to cure, and frequently ends in the worst form of amaurosis. In some persons, owing to idiosyncrasy, mercury cannot be administered, from the distressing effects it produces. Again, when patients have undergone lately one, two, or more courses of mercury, they are unwilling, and sometimes unable, to bear another course of this powerful remedy: in such cases, turpentine should be had recourse to. This remedy was introduced by Hugh Carmichael, Esq., of Dublin: I quote his words on the occasion, from his Observations on the Efficacy of Turpentine in the Venereal and other deep-seated Inflammations of the Eye, published in 1829. "I use the turpentine in this complaint in drachm doses, given three times a day: its disagreeable flavor and nauseating effects I have found best obviated by almond emulsion. This circumstance it is very necessary to attend to, the medicine being so unpleasant that, if its taste be not in some way disguised, it is difficult to depend on patients taking it with the necessary regularity. In the formation of the emulsion, if double the quantity of confection directed in the London Pharmacopœia be employed, (that is, two ounces to the half-pint of water,) it answers the above objects much better: the residuum may be removed by straining.

"With an emulsion so made, the following is the formula I now generally adopt: ℞. Olei Terebinthinæ rectificati, ℥j.; vitellum unius ovi; tere simul, et adde gradatim Emulsionis Amygdalarum, ℥iv.; Syrupi Corticis Aurantii ℥ij.; Spiritus Lavandulæ compositi, ʒiv.; Olei Cinnamoni, guttas tres vel quatuor. Misce, sumat cochl. larga duo ter de die.

"In a few cases it has been necessary to increase the quantity of turpentine to an ounce and a half or two ounces in the above mixture, the other ingredients being proportionally diminished, so that a drachm and a half or two drachms of it may be taken each time; but in general, when administered to the extent directed in this formula, it has very seldom indeed failed, though extensively tried, and in very urgent cases: the instances of its failure shall be presently noticed. The strangury, so frequently induced by the internal use of the turpentine, is obviated by the usual means, flaxseed (the linseed of England) tea, and camphor julep: when very urgent, the medicine may be suspended for a time. The tendency to acidity in the stomach, which it sometimes causes, is relieved by the addition of carbonate of soda to the mixture: ten or fifteen grains to the eight ounces will be sufficient. Some patients have said the taste was further disguised by this addition.

"When the local inflammation is high, and acute pain is present in the eye and side of the head, the abstraction of blood from the temple by cupping, or the more immediate seat of the disease by leeching, may be resorted to: the same practice is adopted when mercury is used. Nevertheless, I have frequently, when these symptoms were very urgent, relied solely on the turpentine mixture, and with the most decided and expeditious relief: indeed, in some instances, where the pain and hemicranium existed as acutely as they are perhaps at any time to be met with, patients have declared they were considerably relieved after they had taken it once or twice, and that its subsequent exacerbations were lessened in a remarkable degree. It is in the former cases I have generally found it necessary to follow up the bleeding by increasing the quantity of the turpentine.

"It is highly necessary to observe, that the condition of the bowels will require attention: the beneficial effects of the medicine appear to be, in certain cases, suspended when constipation is present, and are called forth, as it were, when this is removed."

These are the words of Mr. Hugh Carmichael, and they emanate from a man of great practical experience: they give a clear and concise view of the cases in which turpentine should be employed, of the manner in which it should be administered, and the precautions to be taken in employing it.

It was from the acknowledged influence of turpentine in peritonitis, and the analogy in point of morbid effects between inflammation of the peritoneum and that of the iris: in both cases a serous membrane being engaged, and in both adhesions being produced between surfaces intended to be free, that Mr. Carmichael was led to make use of it in iritis: he deems that it acts in the same manner that mercury does, by exciting the absorbents.

Mr. Guthrie, who has given it an ample trial, finds that "in some cases it has succeeded admirably; in others, it has been of little service; and in some, unequal to the cure of the complaint." Mr. Lawrence has not yet tried this remedy.

From several cases which I have seen, I am inclined to think that turpentine acts by exciting inflammation in the intestinal canal, and more especially the urinary apparatus: the cases which have been most successful, of those which I have seen at least, have been those in which severe strangury has been excited. The degree of counter-irritation thus set up will readily account for the removal of the original disease. I have likewise seen cases in which I found it impossible to excite this irritation, (notwithstanding the violet odour was so powerful that it was complained of throughout the house in which the patient resided, and he was obliged to quit his lodgings accordingly,) and in these no benefit was experienced from its use. I have already said, that it was from the acknowledged influence of turpentine in peritonitis, and the analogy in point of morbid effects between inflammation of the peritoneum and iris, that Mr. Carmichael was induced to try the turpentine: now, as he has taken the example of the use of turpentine in peritonitis to fortify his opinions, I hope I may be allowed the same privilege, of which I shall instantly avail myself, and proceed to ask in what way turpentine acts in curing that inflammation? If we consult the best works on that subject, we shall find it attributed to the irritation produced along the whole alimentary canal, not the intestinal tube only, as it produces nausea, and frequently vomiting: when we consider the immense extent of surface exposed to the action of this medicine in that canal, and recollect the degree of irritation produced by a common purge in that canal, we shall have no difficulty in subscribing to this doctrine; and then reasoning by analogy, if it acts thus in peritonitis, it surely may do so in iritis?

I am also inclined to deny altogether the specific powers of mercury in this or any other disease. If I am right in what I conceive a specific to be, it is a remedy which never fails to cure a disease, and which said disease can never be cured by any other remedy; its action must also be unknown. Now, for a long period of years, this remedy has been deemed a specific in the cure of venereal diseases, as most of you know; but it appears that that disease can be cured, and permanently cured, without one grain of mercury, as shewn by the records of our army surgeons; and, again, certain cases have been exasperated, but more particularly certain symptoms, as the phagedenic ulcer, have been rendered worse, by the employment of mercury: and in this disease (iritis,) I set out with saying that there are many cases in which it would be improper; there are many cases, likewise, in which it has failed: besides, this remedy has been employed, and with success, in many other besides syphilitic diseases, as simple iritis, peritonitis, hepatitis, &c. Having, therefore, as I think, destroyed, in some degree, its pretensions as a specific remedy, I will proceed to state that I think this medicine acts in the same manner, as almost all, if not all, our other medicines do, namely, by exciting counter-irritation. We are told that mercury shews that it is exerting its beneficial effect on the system when it has produced sore mouth, &c., and that, until this occurs, we need not expect lasting benefit from it: this is said to be shewing its *specific effect;* but, with all due submission to the holders of this doctrine, may not mercury cure the disease by the *counter-irritation* it produces in the mouth, salivary, and biliary glands?

But I must leave this subject, and proceed with a few cases I am desirous of detailing, or I shall occupy too much of your time. If the following cases should induce any one to try this remedy (the oil of turpentine,) in those cases in which mercury is inapplicable, I shall deem myself fully rewarded for the time spent in inditing them. The first case which I shall relate will not prove in favor of the Oleum Terebinthinæ, as it failed in curing the complaint.

*Syphilitic Iritis of the Left Eye, attended with a Lichenous Eruption all over the Body, treated by Mercury with Success; then recurring in the Right Eye, and treated with the Oleum Terebinthinæ without any material Advantage; recourse then being had to Mercury, a Cure effected.*

Ann Lawrence, ætat. twenty-three, was admitted October 3d, 1830. About ten days ago, she first perceived a dimness and pain in the left eye, which continued gradually increasing: the iris is much changed in colour; pupil very irregular; the conjunctiva of the lids and ball injected; vision lost; great lachrymation; constant pain; intolerance of light. She says she has never had any venereal complaint; she is married; cannot assign any cause for the disease; suffers from great pain down the spine of the left tibia.—Cucurbitulæ cruentæ ad templum applicentur, et detrahetur sanguis ad ℥xij. ℞. Hyd. Submur. gr. ij.; Pulveris Opii, gr. ¼, fiat pil. in die sumend.

5th. Being pressed on the subject, acknowledges that she has gonorrhœal discharge, which her husband communicated to her: it first appeared about twelve weeks since, and she has not entirely got rid of it yet. On examination, the whole body was covered with a lichenous eruption. The pain in the eye is much diminished since the cupping; the appearance of the eye not materially altered.—Cont. medicamenta.

7th. The inflammation of the iris is lessened; vision somewhat improved; pain not so great; pupil still very irregular and contracted; the mouth is becoming sore from the pills: to take them once a day only.

10th. A very decided change for the better: iris nearly free from inflammation; pain very slight; is free from lachrymation; cannot bear the light; mouth still sore; bowels open.—℞. Hydr. Submur. gr. ij.; Pulv. Opii, gr. ¼. Rept. pil. bis die sumend.

12th. Still continues improving.—Rept. pil. Instilla gutt. Belladonnæ.

19th. Vision improved; no pain; inflammation almost gone; general health good; pupil more regular; eruption much the same. —Rept. pil. Instillet Belladonna.

21st. Going on well.—Pergat.

24th. The mouth is very sore from the pills: to take them but once a day. The appearance of the eye is quite natural; vision still indistinct; eruption gradually fading away.—Rept. guttæ.

Nov. 2d. Has been absent from London for some days: the eye is nearly well; a very small degree of irregularity of the pupil still remains; vision rather dim; eruption continues fading.—Rept. instillatio Solut. Belladonnæ, et persistet in usu pil.

This patient got quite well, and, though some slight degree of dimness remained, yet it was daily disappearing.

20th. She returned today with well-marked iritic inflammation of the right eye, which she first perceived about three days ago: the pupil is somewhat irregular; the conjunctiva very much inflamed; complains of very severe pain in the eye, and also in the head, accompanied with intolerance of light; the vision is nearly lost; the eruption has disappeared.—App. Cucurb. cruent. ad ℥x. temp. dextr. ℞. Hyd. Submur. gr. vj. fiat pil. h. s.s, ℞. Sulph. Magnes. ℥ss. cras mane sumend. Sumat Ol. Terebinth. ʒi. ter in die.

23d. The eye appears worse today: the inflammation of the iris greater; pupil more contracted and irregular; vision entirely gone; bowels open.—Contin. Ol. Terebinth.

24th. Complains of great pain in the head; great lachrymation; tears hot; inflammation increased.—Venæsectio ad ℥xvj. Rept. Ol. Terebinth.

25th. The bleeding has relieved her head; the pain in the eye is decreasing.—Pergat.

26th. Very little improvement; pain in the eye not constant. —Rept.

28th. Much the same.

30th. The inflammation of the iris is still very great; pupil more contracted; complains still of pain in the head; pulse feeble; bowels open.—Rept. Oleum.

Dec. 4th. Says she cannot keep the turpentine on her stomach: is directed to leave it off. No material change in the eye.—℞. Hydr. Submur. gr. ij.; Pulveris Opii, gr. ¼. M. fiat pil. c. conf. ter in die sumend.

6th. The eye is still extremely painful; iris continues greatly inflamed; great lachrymation; severe pain in the head; tongue white and furred; bowels open.—Rept. pil.

8th. Much worse today: the inflammation of the iris on the increase; pupil more contracted and irregular; very severe pain in the head and eye. Mr. Guthrie ordered her to rub in the Ung. Hydrargyri on the temple; to be cupped to ℥xij., and to take the pills every two hours.

11th. Greatly improved this morning: iris not so much inflamed; the cupping has removed the pain; pupil still contracted and irregular.—Rept. pil. Instillet in oculo gutt. Sol. Belladon.

13th. No pain in the eye or head; inflammation much lessened; vision rather improved; pupil very irregular. The belladonna drops have been applied yesterday and today.—Rept. pil. ter in die.

14th. Vision continues improving: inflammation of the conjunctiva nearly gone; no pain; inflammation of the iris disappeared; pupil still contracted and irregular.—Persistet in usu instillationis guttæ Belladonnæ.

This case was communicated by an highly intelligent and esteemed friend, who has since left this quarter of the globe.

With the permission of the Society, I shall proceed to make a few remarks on this case. The long duration of the second attack, its resisting the turpentine, the exacerbation of the symptoms during the use of mercury in the form of pill, and their mitigation when that remedy was exhibited in the form of ointment, are prominent features in this case, and render it, in my opinion, highly interesting. The occurrence of iritis so soon after a course of mercury, as in the second attack, might favor the belief in the mercurial iritis, were it not that it resisted the turpentine, and yielded to mercury: unless, indeed, we allow mercury to possess the powers attributed to Achilles' spear, that it can cure the wounds it makes. Medical theories are very troublesome: if we give too much mercury in the treatment of primary symptoms of syphilis, we are told by one party, if secondary symptoms supervene, that they are mercurial; if, however, the course was not sufficiently extended to please another party, then, forsooth, the disease was not eradicated, and we must begin *de novo*. Whom or what are we to believe? Either mercury does cause secondary symptoms, and syphilis does not, or *vice versâ;* or else, what is more perplexing still, they each produce secondary symptoms, exactly similar? We are told to "trust to experience:" but to what experience is the young practitioner to trust? Surely not his own, for he is presumed to have none.

But I have wandered from my subject, and must resume it, which I proceed to do by relating the next case, which is one of

*Syphilitic Iritis of the Left Eye, consequent on Chancre and Gonorrhœa, treated successfully with the Oleum Terebinthinæ.*

Thomas Calvers, ætat. twenty-three, admitted June 6th, 1831; is suffering from severe inflammation of the iris of the left eye. In October last he had a chancre, and was salivated: this was cured in about a fortnight. About a week before the chancre disappeared, a discharge *per urethram* came on, attended with severe scalding pain on voiding his urine: the discharge continued some time after the scalding had been removed. When the running had stopped about a fortnight, the throat became inflamed and ulcerated: this was in last February; it has continued painful and inflamed ever since.

About a month ago the eye inflamed, and got gradually worse: leeches were applied, which gave him considerable relief. The iris and conjunctiva are very much inflamed; pupil contracted; throat in a state of ulceration.—℞. Ol. Terebinth. ʒj. ter in die sumend.

10th. He is free from pain in the eye and any sensation of itching. His eye felt better on the evening of the 9th; the throat is better; the urinary organs are slightly affected.—Rept. Oleum.

13th. On the 11th, there was a slight degree of pain in the eye, which went off in the evening. On the 12th, the pain was more severe. He complains today of a slight pain in the eye; the iris is somewhat irregular; a few spots are making their appearance on the back; throat more painful; there is pain in the loins, and a slight degree of pain on voiding his urine.—Cucurb. cruent. ad ℥x. tempori. Rept. Ol. Terebinth. Instillat. gutt. Belladon. The belladonna partially dilated the pupil, and shewed plainly its irregularity.

15th. Scarcely any pain in the eye. Suffers great pain while at stool, with slight scalding on passing his urine.—Pergat.

17th. The inflammation of the iris has disappeared; the bowels are violently acted upon by the terebinthinate; no pain, but slight dizziness.—Omit the turpentine, and let him drink freely of linseed tea.—Magn. Sulph. ʒj. mane sumend.

18th. The inflammation has recurred, attended with pain, &c. —Resume the use of the turpentine.

19th. The right eye is slightly inflamed; the Infus. Lini has removed the scalding.—Rept.

22d. Complains of headach.—C. C. ad ℥x. temp. Magnes. Sulph. ℥j.; Ant. Tart. gr. ij. nocte sumend. To omit the Terebinthinate.

The inflammation of the iris, in a day or two after this date, disappeared. The further report, in my notes, states that the constitutional symptoms became more severe, but were finally cured. During treatment, he had frequent relapses of conjunctivitis, which were, however, speedily cured. This case was communicated to me by Mr. Nice.

I have notes of two other very interesting cases; but, as the paper is already very long, I dread trespassing on the time of the Society, and shall only add, that they well exemplified the benefits derived from the use of this medicine.

36, *Tavistock street, Covent Garden.*

